# A Miniature Probe for Ultrasonic Penetration of a Single Cell

**DOI:** 10.3390/s90503325

**Published:** 2009-05-06

**Authors:** Ting Wu, Zhaoying Zhou, Qun Wang, Xing Yang, Mingfei Xiao

**Affiliations:** 1 MEMS Lab, Department of Precision Instruments & Mechanology, Tsinghua University, Beijing 100084, P.R. China; E-Mails: zhouzy@mail.tsinghua.edu.cn; yangxing@mail.tsinghua.edu.cn; xiaomf03@mails.tsinghua.edu.cn; 2 School of Information Technology, China University of Geosciences (Beijing), Beijing 100083, P.R. China; E-Mail: qunw@cugb.edu.cn; 3 State Key Laboratory of Precision Measurement Technology and Instruments, Tsinghua University, Beijing 100084, P.R. China

**Keywords:** Ultrasonic penetration, FEM, ultrasonic probe, cell, generator

## Abstract

Although ultrasound cavitation must be avoided for safe diagnostic applications, the ability of ultrasound to disrupt cell membranes has taken on increasing significance as a method to facilitate drug and gene delivery. A new ultrasonic resonance driving method is introduced to penetrate rigid wall plant cells or oocytes with springy cell membranes. When a reasonable design is created, ultrasound can gather energy and increase the amplitude factor. Ultrasonic penetration enables exogenous materials to enter cells without damaging them by utilizing instant acceleration. This paper seeks to develop a miniature ultrasonic probe experiment system for cell penetration. A miniature ultrasonic probe is designed and optimized using the Precise Four Terminal Network Method and Finite Element Method (FEM) and an ultrasonic generator to drive the probe is designed. The system was able to successfully puncture a single fish cell.

## 1. Introduction

Single cell membranes are selective in that only water, ions and small molecules are able to pass through easily, while the exchange of other materials is hindered. Since many important biochemical reactions happen inside the cell, numerous techniques have been developed for the transmission of exogenous materials through cell membranes. These techniques include electroporation [1-2], liposome transfection [3], microinjection [4] and so on. In 2005, Obataya [5] was able to successfully penetrate a cell membrane in atomic force microscopy (AFM) via a Si needle. The same year, Kouklin [6] made a bundle of carbon nanotubes penetrate into a cell. The patch clamp technique [7], which won the inventor the Nobel Prize in 1991, further simplified single cell penetration and organelle analysis. However, limited by inaccuracy of cell displacement and shape, the microcapillary may cause fatal damage to a cell, reduce the cell's activity, or bring about some other undesirable consequences as a result of a cell's penetration [8]. There are some disadvantages in using static force, including a low possibility of success rate, and the destruction of the cell's normal structure due to the large deformation of the cytoskeleton [9]. In this paper, a new ultrasonic resonance driving method is introduced to investigate cell penetration. Compared with manual micro-operation, tremendous force and acceleration can be provided by ultrasonic driving, through which the phenomena resulting from the use of a static force can be avoided. Based on these characteristics, the new method primarily works both on plant cells, which have a rigid wall or oocytes with springy cell membranes, and achieves the injection of exogenous materials and the attraction of intracellular components.

Power ultrasound is applied widely. For example, it is used in precise operations such as neurosurgery, arthrosis surgery and orthopedic surgery. When the ultrasonic probe we designed is working on resonance, the energy of an ultrasonic longitudinal wave is centralized on a smaller cross- sectional area of an ultrasonic horn. The centralized energy provides tremendous force and acceleration, and efficient puncture is thus achieved. It has been reported that a force of no more than 10 nN is required when a normal pyramidal AFM tip is used in order to penetrate blood and fibroblast cell surfaces [10]. According to the second law of Newton, driven by a 40 kHz ultrasonic force, a probe tip which has an average diameter of 5 μm and length of 10 μm, can produce a peak puncture force of 67 nN, which is far higher than the force needed in practice. We therefore decided to make use of these characteristics to research single cell penetration. However, we were aware that ultrasonic cavitation may cause the cell's disruption [11]. Cavitation is the creation and oscillation of small gas bubbles that can violently implode if sufficient ultrasonic pressure is applied. In our design, use of a nanoprobe and micro amplitude have guaranteed that the ultrasonic cavitation energy is lower than the secure threshold in the penetration position, so any injuries to the cell can be avoided.

In this study, an ultrasonic probe was designed and optimized. When model analysis and harmonic analysis of the ultrasonic probe were made by using ANSYS (FEM software), the parameters of the probe, such as amplitude, precise longitudinal vibration mode and resonance frequency, can all be calculated by Finite Element Analysis (FEA). An ultrasonic signal generator was designed to drive the probe, and a cell penetration system was established to perform experiments on fish cells. The cell penetration results indicated that the system was usable and effective.

## 2. Design and Fabrication

### 2.1. Experimental System Design

**Figure 1.**
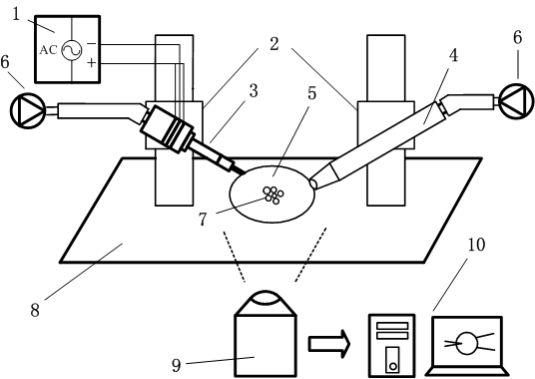
Sketch of the experimental system for single cell penetration.

The whole experimental system is shown as Figure 1. It has three functions including attracting and immobilizing a single cell, clamping and controlling the ultrasonic probe and outputting a powerful, high frequency sine signal. The whole system was set up on a vibration isolation platform so as to be shielded from external disturbances. Four subsystems are included: a driving component, an actuation component, a micro-manipulation component and a real-time observation and measurement component.

The driving component transported electric energy to an ultrasonic probe to drive the piezoelectric material. The core component was an ultrasonic signal generator (1).

The ultrasonic signal generator (1) was designed for and implemented in this study. It produces a sine wave signal, whose frequency can be specified from 10 Hz to 999 kHz. The maximum signal amplitude can reach 400 V. It has the function of automatically regulating the frequency of the ultrasonic probe. The actuation component includes an ultrasonic probe (3) and a single cell attracting device (4). The ultrasonic probe (3), equipped with a nano tungsten needle, is the core component of the whole system. It was designed and optimized in this study.

The single cell attracting device (4) optimized for single cell catching was designed based on a medical transfusion system. Cells (7) were placed in the glass service (5). A cell may be attracted on the tip of a glass capillary and be moved to any positions where it is expected to be.

The micro-manipulation component consists of two three-dimensional micro-manipulators (2) and two pressure-conditioning devices (6). The smallest three-step movement the micro-manipulators can achieve in X, Y, or Z directions is one micron; they are set up with optical fixtures for clamping the ultrasonic probe (3) and the attracting device (4). The pressure-conditioning devices (6) control the attraction intensity of the attraction device (4) and the output of the probe (3).

The real-time observation and measurement component consists of a biological inverted microscope (9), a high-speed camera, a computer (10) and its corresponding software. All devices were installed on a vibration isolation platform (8) so that they were shielded from external disturbance.

### 2.2. Ultrasonic Generator Design and fabrication

**Figure 2.**
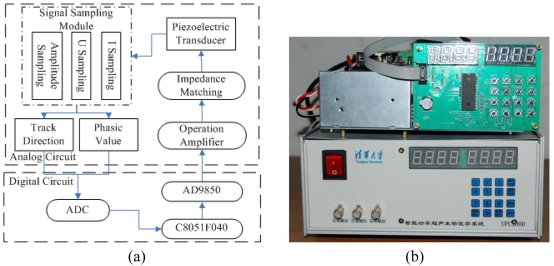
(a) Block diagram of ultrasonic generator. (b) A picture of ultrasonic generator.

A series of experiments attempting to puncture a single cell were conducted, and they showed that when the ultrasonic probe was operated, its working characteristics would change. Compared with the unloaded condition the working frequency changed and the bandwidth of the admittance of the real part and radius of the admittance circle changed accordingly. In order to ensure that the probe is working in resonance and the output acoustic impedance matches the loading impedance, an automatic regulator had to be designed to regulate the frequency of the ultrasonic probe. A new ultrasonic generator, which consisted of a DDS synthesizer, a power amplifier and an automatic frequency regulator, was manufactured to improve the working stability of the system. Figure 2(a) presents the block diagram of its structure, and (b) is a picture of the ultrasonic generator be developed.

A frequency control signal chip (AD9850), using advanced direct digital synthesis (DDS) technology, generated a pure, frequency/phase-programmable, analog output sine wave. A switch capacitor filter (MAX268) was used to filter the signal from AD9850. The filter center frequency changed with the input frequency. A high-voltage, high-precision MOSFET op-amp (PA85) was the key component in the power operation amplifier. The amplifier can achieve the linear amplify function and adjust the frequency. Signal sampling module consisted of amplitude sampling, voltage sampling and current sampling. A micro-controller (C8051F040) was used to sample the input and output of the voltage and current. The frequency and amplitude of the ultrasonic probe were controlled by the micro-controller.

### 2.3. Ultrasonic Probe Design and fabrication

The ultrasonic probe consists of a transducer, an ultrasonic horn and a nano needle. The transducer is the key part, which utilizes piezoelectricity and transforms electrical energy into mechanical vibrations. The ultrasonic horn, which has the effect of amplification and coupling, is used to impel the needle. The nano tip of the ultrasonic probe is made of tungsten, which was etched by an electrochemical method [12].

The frequency, displacement and acceleration of the ultrasonic probe are necessary to be considered in order to ensure the penetration of a cell. The acceleration can be expressed as:

(1) $$a(t)=-4\pi^{2}f^{2}A\sin(2\pi ft+\varphi )$$

where, *f, A, φ* are the frequency, amplitude and initial phase respectively. The breakdown acceleration threshold of a soft tissue is about 50,000 g (g = 9.8 m/s^2^). If the frequency and the amplitude were 40 kHz and 7.7 μm respectively, the value of acceleration would exceed the threshold value of 50,000 g.

**Figure 3.**
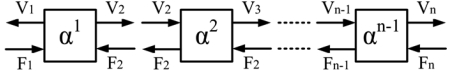
Four-terminal network principle.

The Precise Four Terminal Network Method and Finite Element Simulation, with which the ultrasonic probe is designed, are introduced as follows: an ultrasonic transducer was schemed out by the four-terminal network method [13-15], which is a novel design method. Its basic principle is that a single level ultrasonic horn can be regarded as an equivalent mechanical four-terminal network. Every adjacent level is connected in series (see Figure 3).

An arithmetic expression of n level network is shown in (2):

(2) $$\left[\begin{array}{c} F_{n}\\ V_{n} \end{array}\right]=\left[\begin{array}{cc} \alpha_{11}^{n-1} & \alpha_{12}^{n-1}\\ \alpha_{21}^{n-1} & \alpha_{22}^{n-1} \end{array}\right]\;\left[\begin{array}{c} F_{n-1}\\ V_{n-1} \end{array}\right]=\left[\begin{array}{cc} \alpha_{11}^{n-1} & \alpha_{12}^{n-1}\\ \alpha_{21}^{n-1} & \alpha_{22}^{n-1} \end{array}\right]\ldots \left[\begin{array}{cc} \alpha_{11}^{1} & \alpha_{12}^{1}\\ \alpha_{21}^{1} & \alpha_{22}^{1} \end{array}\right]\;\left[\begin{array}{c} F_{1}\\ V_{1} \end{array}\right]=\left[\begin{array}{cc} \alpha_{11} & \alpha_{12}\\ \alpha_{21} & \alpha_{22} \end{array}\right]\;\left[\begin{array}{c} F_{1}\\ V_{1} \end{array}\right]$$

where *F, V* are the force and acceleration of each level of ultrasonic horn, respectively. *α* is a shape parameter related to the horn's geometrical shape, material parameters, dimensions and resonance frequency. The two ends of the ultrasonic horn are not restricted. So *F*_1_=*F_n_*=0. The equation (2) is simplified to (3):

(3) $$\left[\begin{array}{c} 0\\ V_{n} \end{array}\right]=\left[\begin{array}{cc} \alpha_{11} & \alpha_{12}\\ \alpha_{21} & \alpha_{22} \end{array}\right]\;\left[\begin{array}{c} 0\\ V_{1} \end{array}\right],\;0=\alpha_{11}\cdot 0+\alpha_{12}\cdot V_{1}$$

In equation (3) *V*_1_≠0, *α*_12_=0. Before an ultrasonic probe is fabricated, the parameters which include the diameter and length of each layer and the value of harmonic frequency must be given. If the harmonic frequency and the diameter of each layer were estimated in advance according to the given amplitude ratio and other requirements, and some lengths of the ultrasonic horn were also confirmed and only one parameter was unknown, and this unknown parameter can be obtained from equation (3).

**Figure 4.**
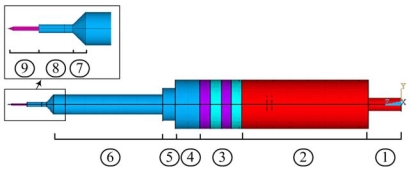
Schematic of ultrasonic probe.

Based on the theory above, a Precise Four Terminal Network computation software package was developed. With the software, the vibration resonant frequency can be obtained. If the resonant frequency is known, the size of one layer can be solved. The final design scheme is shown in Figure 4, and materials and parameters are listed in Table 1.

In this design, the echeloned ultrasonic horn was adopted to achieve higher amplitude magnification. A conic transition region was introduced to the transformable section to protect the probe against stress centralization. The tungsten needle was etched by an electrochemical method, and a tungsten tip of nanometer apex dimension was produced. Electrodes were placed on the two surfaces orthogonal to the polarization axis.

The designed amplification ratio of the amplitude was 10. The resonance frequency was 40 kHz. With high transfer efficiency of electric-mechanic and low heat generation coefficient, the piezoelectric material (PZT-8) is very suitable for miniature actuators. The back end of the probe was made of brass (which has high acoustic impedance) in order to reduce the length and to increase the energy transfer to the front part. The front end of the probe was made of titanium alloy, which is stiff with low density. It was not easy to deform into a small diameter. The diameter of the tungsten tip was designed to be only 5 μm in order to easily puncture a cell (the diameter of a cell being 100-500 μm).

FEM software (ANSYS 9.0) was used to evaluate status, resonant frequency and displacement of an ultrasonic probe through the model analysis and the harmonic analysis. The finite element model was established using the structural parameters (see Table 1). Properties of brass, titanium alloy and tungsten, which contained Young's modulus, Poisson's ratio, density, were referred to in the table of material properties. Setting properties of piezoelectric material was the key. A piezoelectric model required the permittivity (or dielectric constants) matrix [*ε_r_*], the piezoelectric matrix [*e*], and the stiffness matrix [*c*] to be specified as material properties. The PZT8 material we used was polarized along the Z axis. The constitutive matrices (4) ∼ (6) were as shown below:

(4) $$[{ɛ}_{r}]=\left[\begin{array}{ccc} 7.97 & & \\ & 7.97 & \\ & & 5.13 \end{array}\right]\times 10^{-9}$$

(5) $$[e]=\left[\begin{array}{ccc} 0 & 0 & -4.1\\ 0 & 0 & -4.1\\ 0 & 0 & 14.0\\ 0 & 10.3 & 0\\ 10.3 & 0 & 0\\ 0 & 0 & 0 \end{array}\right]$$

(6) $$[c]=\left[\begin{array}{cccccc} 14.9 & 8.11 & 8.11 & 0 & 0 & 0\\ & 14.9 & 8.11 & 0 & 0 & 0\\ & & 13.2 & 0 & 0 & 0\\ & & & 3.40 & 0 & 0\\ & & & & 3.13 & 0\\ & & & & & 3.13 \end{array}\right]$$

**Figure 5.**
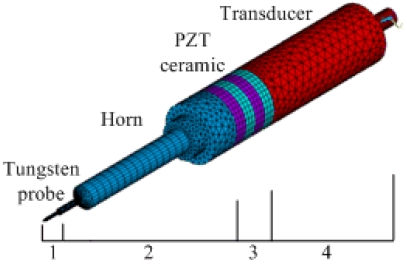
FEM grid model of ultrasonic probe.

Piezoelectricity is the coupling of structural and electric fields, which is a natural property of some materials such as quartz and ceramics. Piezoelectric thin films were modeled using 3-D Coupled-Field Solid5. Since the model was complex, the regular parts were meshed to the hexahedral elements by sweeping generation and the irregular parts were meshed to the tetrahedron elements by automatic generation. The FEM grid model is shown in Figure 5.

**Figure 6.**
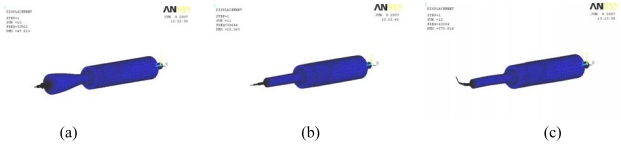
Three types of vibration modes. (a) 37801Hz. (b) 39644Hz. (c) 43264Hz.

Model analysis was performed using the Block Lanczos method. Figure 6 shows three modes, all of which have a frequency of about 40 kHz. Among them, the second mode at 39.644 kHz was the longitudinal vibration that we expected.

**Figure 7.**
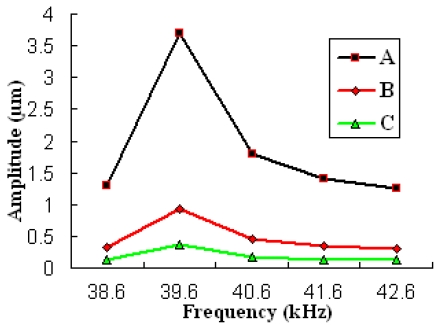
Amplitude & Frequency by FEM in different points. A-The tungsten needle (found in section 9 of Figure 4). B-The second-class amplitude amplified part of the horn (found in section 6 of Figure 4). C-The end segment of the horn (found in section 3 of Figure 4).

The Jacobi Conjugate Gradient (JCG) solver was selected in full harmonic analysis. The displacement of all nodes could be obtained in post-processing analysis. We took three different points A, B and C, which are indicated by sections 9, 6 and 3 in Figure 4 respectively. The amplitudes of different points when the input voltage was 60 V are shown in Figure 7. The amplitude of each section was different, and the points in the same section had the same amplitude.

The energy transfer relationship of the horn can be found in Figure 7. The amplitude of the tip (A) was 3.7 μm, and the amplitude of the piezoelectric ceramic (C) (the end segment of the horn) was 0.39 μm under the frequency of 39.644 kHz. The amplitude ratio was 9.5, which was close to the design value of 10. From the result of the FEM, the axial amplitude of the probe was 3.7 μm, and the radial amplitude was 1.38×10^-2^ μm by a driving voltage of 60 V. We calculated that the ratio of the vibration velocity in lateral direction to that in longitudinal direction was about 0.0037.

**Figure 8.**
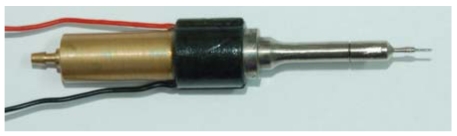
A picture of the ultrasonic probe

Based on the above calculations, an ultrasonic probe with a nano tungsten needle was fabricated as shown in Figure 8. The cell penetration experiments were done using this probe.

## 3. Experimental Results

The amplitude of the probe was measured by a laser amplitude tester (Polytec-700, Polytec, Germany). When the driving voltage was 60 V, we tested the amplitude of the tip and the piezoelectric ceramic, and the experimental results were 0.43 μm and 3.9 μm respectively. The amplitude ratio was 9.1. A dynamic signal analyzer (SR785, Stanford, USA) was used to measure the resonance frequency of the probe, and the experimental result of 39.2 kHz was obtained. Four parameters in different conditions are shown in Table 2.

In Table 2, the theoretical results of the frequency and amplitude ratio were obtained using Four Terminal Network Method and simulation results were from FEM using ANSYS. The amplitudes of piezoelectric ceramic and tip were not figured out by the Four Terminal Network Method directly. The theoretical results of the amplitudes were calculated by piezoelectric constant of the material PZT-8, the external field intensity, and the magnification ratio of the amplitude. The amplitude of the tip and the piezoelectric ceramic were 4 μm and 0.4 μm respectively. The experimental results were measured by the instruments. Compared with experimental result of the resonance frequency, the Precise Four Terminal Network Method had an error of 2 %. Because mechanical loss was not taken into consideration, the result of FEM had an error of 1.1 %, compared with the experimental result of 39.2 kHz.

**Figure 9.**
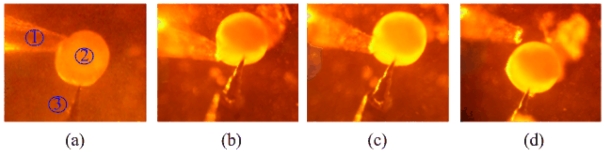
Process of single cell penetration by the ultrasonic probe. (a) Tip moving up to the cell. (b) The instant of penetration. (c) Probe within the cell. (d) Cell released.

This experiment system was successful in puncturing a fish cell. Under the microscope and CCD camera, the full process of single cell penetration by the ultrasonic probe was recorded. The target cell was a fish egg with a diameter of 300 μm. The driving voltage to the ultrasonic probe approximated to 110 V. The amplitude of the tip was larger than 7.7 μm. Figure 9(a) shows the tip is moving up to a cell. Figure 9(b) shows the instant of ultrasonic penetration. The tip of the probe within a cell is seen in Figure 9(c). After the cell was released from the cell catcher, it was able to follow the probe and move within a small range [see Figure 9(d)].

From the observation of a cell penetration, no deformation of the target cell was obvious, and no tissue disruption or cytoplast leakage phenomena occurred during the experiment. The internal damage we were concerned about could mainly come from two sources: the needle and heat energy. The diameter of the solid needle was smaller than the common glass needles used in traditional micro-manipulation. Moreover, the needle was made of tungsten, which is biologically compatible, so any potential harm from the microneedle could be ignored. In numerous experiments, the instantaneous ultrasonic penetration captured by high speed photography (20,000 fps) was finished within 100∼300 ms, depending on the kind of cells, so we conclude that any heat damage could be ignored too so overall the operation was rapid, convenient, precise and harmless.

**Figure 10.**
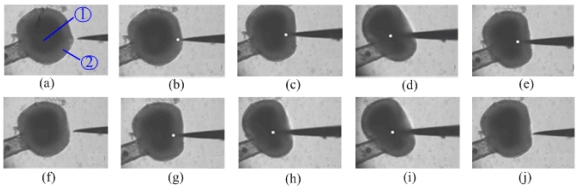
Process of single cell penetration without ultrasonic driving. (a ∼ d) Tip moving up to cell for the first time. (e ∼ f) Tip moving out. (g ∼ i) Tip moving up to cell for the second time. (j) Tip moving out.

We carried out another experiment using static force to verify the effectiveness of the ultrasonic penetration. The single cell penetration process without ultrasonic driving was shot by a high-speed camera (Fastcam SA1, Photron, Japan), and some images are shown in Figure 10. There are four steps: the tip moving up to cell for the first time are shown in Figures 10(a ∼ d); the tip leaving and the cell without any harm are shown in Figures 10(e ∼ f); the above steps were repeated and the same result was obtained, as displayed in Figures 10(g ∼ j). The tip of the probe is surrounded by the distorted cell. Therefore, its position was marked using the white dot.

From the cell penetration observations, the zona pellucida is apparently distorted in Figures 10(d) and (h). As the displacement of the tip increased, deformation of the target cell was obvious. Therefore, the cell organelle was damaged due to the stress suffered by the movement of the tip. The displacement of the micro needle exceeded the original radius of the cell, but the cell was not punctured successfully. Thus, introduction of ultrasonic driving method to bio-micro-manipulation is necessary and effective.

## 4. Discussion and Conclusions

The acceleration of the probe must exceed the threshold value of 50,000 g in order to ensure the penetration of a cell, so the appropriate frequency and amplitude were calculated in the paper. The test results of the micro-ultrasonic probe were consistent with the results of the theoretical calculation and Finite Element Analysis. The experiments indicated that the probe was could be successfully applied in the penetration of a single cell.

The damage from the heat energy was the main problem that was considered originally. The problem was solved through the optimum design of the ultrasonic probe and output signal matching of the ultrasonic generator. The hatching result of the eggs would be the most conclusive evidence on whether any damage happened inside the cell, but currently we do not have facilities in our laboratories to perform such experiments. We hope to be able to provide additional evidence in the future.

A new technology typically needs to go through a long period of time from proposal to application, especially in biomedical research. Compared with other parallel techniques, such as electroporation and gene transfer, the ultrasonic driving method introduced in this paper was proven to be an effective supplement to traditional micro-manipulation. This ultrasonic penetration technique possesses extensive potential applications in nanobiology and nanomedicine. We consider that the ultrasonic micro-manipulation technique can be applied to single cell analysis, nuclear transplantation, inner cell information analysis, clone and drug delivery, etc. Furthermore, the effectiveness of the traditional micro-operation is enhanced.

## Figures and Tables

**Table 1.** Design parameters of ultrasonic probe*

**Number**	1	2	3	4	5	6	7	8	9
**Material**	brass		PZT-8	titanium alloy					tungsten
**Diameter (mm)**	3	10	10	10	6.5	4	4∼1	1	0.5
**Length (mm)**	6.8	25.5	8.5	5	2.5	22	1.5	4	3.7

* Numbers refer to Figure 4

**Table 2.** Parameters comparing the theoretical, simulation and experimental results.

**Parameters**	**Theory**	**Simulation**	**Experiment**

Frequency (kHz)	40	39.644	39.2
Amplitude ratio	10	9.5	9.1
Piezoelectric amp. (μm)	0.4	0.39	0.43
Tip's amplitude (μm)	4	3.7	3.9
